# Inhibition of liver cancer cell growth by metabolites S-adenosylmethionine and nicotinic acid originating from liver progenitor cells

**DOI:** 10.1007/s00535-025-02226-y

**Published:** 2025-02-28

**Authors:** Wen-Ming Liu, Cai-Yang Chen, Hong-Qian Ma, Qiu-Qiu Zhang, Xu Zhou, Yu-Ling Wu, Wei-Jian Huang, Xiao-Shu Qi, Yu-Xin Zhang, Dan Tang, Han-Yong Sun, Hong-Ping Wu, Ying-Fu Jiao, Zhi-Ying He, Wei-Feng Yu, He-Xin Yan

**Affiliations:** 1 Department of Anesthesiology, Renji Hospital, Shanghai Jiaotong University School of Medicine, Shanghai, China; 2 Institute for Regenerative Medicine, Shanghai East Hospital, School of Life Sciences and Technology, Tongji University, Shanghai, China; 3 Key Laboratory of Anesthesiology (Shanghai Jiao Tong University), Ministry of Education, Shanghai, China; 4 Shanghai Engineering Research Center of Peri-Operative Organ Support and Function Preservation (20DZ2254200), Renji Hospital, Shanghai, China; 5 Department of Liver Surgery, Renji Hospital, Shanghai Jiaotong University School of Medicine, Shanghai, China; 6 Department of Laboratory Medicine, Eastern Hepatobiliary Surgery Hospital, Shanghai, China

**Keywords:** Human hepatocyte-derived liver progenitor-like cells, Hepatocellular carcinoma, Mitochondrial dysfunction, Notch1 and JAK1/STAT3, Metabolite

## Abstract

**Background:**

Hepatocellular carcinoma (HCC), the most common form of liver cancer, presents a challenging malignancy with scarce treatment options. Liver progenitor cells (LPCs) play a pivotal role in both liver regeneration and the progression of liver cancer, yet the specific functions of LPCs from different origins in liver cancer remain to be fully elucidated.

**Methods:**

We explored the liver progenitor-like cells derived from human hepatocytes (HepLPCs) on the proliferation of HCC both in vitro and in vivo. The mitochondrial function was assessed through electron microscopy and functional experiments. Transcriptomic sequencing and western blot unveiled the fundamental mechanisms at play, whereas metabolomic sequencing pinpointed crucial effector molecules involved in the paracrine secretion of HepLPCs.

**Results:**

By employing a co-culture system of HepLPCs and HCC cells, we found that HepLPCs markedly inhibited HCC growth by prompting mitochondrial dysfunction, which further led to the co-inhibition of the Notch1 and JAK1/STAT3 signaling pathways through paracrine actions involving S-adenosylmethionine (SAM) and Nicotinic acid (NA).

**Conclusions:**

This study has uncovered that HepLPCs have a suppressive influence on the proliferation of HCC cells. This is achieved through the impairment of mitochondrial function and the inhibition of key signaling pathways, namely, Notch1 and JAK1/STAT3, which are critical drivers of cancer progression. The secretion of the metabolites SAM and NA by HepLPCs appears to be instrumental in mediating these effects. These findings provide a solid foundation for identifying new therapeutic targets and clarifying the mechanisms through which HepLPCs can be harnessed to effectively treat HCC.

**Supplementary Information:**

The online version contains supplementary material available at 10.1007/s00535-025-02226-y.

## Background

Hepatocellular carcinoma (HCC), the most common type of primary liver cancer, is a global health concern and the main cause of cancer**-**related death worldwide [1, 2]. Despite notable improvements in cancer prevention, diagnosis, and management over the past few decades [3, 4], a great proportion of patients with HCC are still diagnosed at advanced stages, leaving them with limited therapeutic options, such as liver resection, tissue ablation, and liver transplantation. Compounding these challenges are the critical shortage of organs for transplantation and a high rate of cancer recurrence, further complicating the clinical management of HCC [5, 6]. Therefore, understanding HCC's molecular underpinnings and developing novel therapeutic agents are crucial for slowing disease progression and enhancing patient outcomes.

Liver progenitor cells (LPCs) have the ability to evolve into hepatocytes, cholangiocytes, and hepatic stem cells [7]. By employing small molecule reprogramming technology to induce the dedifferentiation of primary hepatocytes back into LPC, we have recently recapitulated the LPC regeneration in vitro, allowing for the large-scale expansion of hepatocyte-derived liver progenitor cells in culture. These cells can then be re-differentiated into mature and functional hepatocytes. We have termed these cells “Human hepatocyte-derived liver progenitor-like cells (HepLPCs)” [8, 9]. Encouraging LPC regeneration shows great potential for treating liver diseases, such as cirrhosis, hepatitis, and liver cancer [10].

Owing to their inherent hepatic properties, HepLPCs present a promising avenue for treating liver injuries. Our research has yielded two significant findings within this field. Initially, we found that encapsulating HepLPCs within alginate microcapsules significantly enhanced the survival rates of mice suffering from acute liver injury [11]. Furthermore, the application of HepLPC-based in vitro bioartificial liver therapy demonstrated an impressive survival rate in pigs with acute liver injuries, underscoring its potential viability for clinical use [12]. This suggests that HepLPCs might also manifest their therapeutic benefits through paracrine signaling mechanisms. Consequently, it becomes particularly compelling that the regulatory influence of HepLPCs on liver cancer still remains to be fully elucidated.

In this study, we investigated the regulatory impact of HepLPCs on HCC and elucidated the underlining mechanisms.

## Methods

### Human cell lines

The human HCC cell lines, including MHCC97H, MHCC97L, Hep3B, HCCLM3, L02, and HepG2, were purchased from the Cell Bank of the Chinese Academy of Sciences (Shanghai, China). Huh7 and PLC/PRF/5 were provided by the Shanghai Cancer Institute & Department of Renji Hospital (Shanghai, China). HCC cells were cultured in Dulbecco’s Modified Eagle Medium (DMEM) with 10% fetal bovine serum, glutamine, and penicillin/streptomycin (Gibco) at 37 °C and 5% carbon dioxide (CO_2_). Cryopreserved HepLPCs were plated on a Matrigel-coated (Corning) culture dish (NEST Biotechnology) at 0.5–2 × 10^4^ cells/cm^2^ and cultured in transition and expansion medium (TEM) as previously described [11]. HepLPCs were immortalized by expressing HPV E6/E7 to achieve expansion without growth arrest.

A PCR-based technique was employed to ensure the absence of mycoplasma contamination. In addition, the authenticity of all cell lines was verified using short tandem repeat (STR) profiling.

### EdU incorporation assay

The BeyoClick™ EdU cell proliferation kit with Alexa Fluor 488 (Beyotime Biotechnology, Shanghai, China) was applied for the EdU incorporation assay. This experiment was performed following the manufacturer’s protocols.

### Plate colony formation assays

Cells were seeded into 12-well plates (1 × 10^3^ cells per well) and cultured in the presence of HepLPC–CM or drugs. For each cell line, cells cultured under different conditions for 7 days were fixed with 4% paraformaldehyde. Afterwards, cells were stained with 0.1% crystal violet (in water). Finally, the excess staining solution was washed off with phosphate-buffered saline (PBS) and cells were dried before taking photos.

### Hepatocellular carcinoma tissue-derived organoids culture

The live cell isolation and organoid establishment and expansion were conducted following established protocols [13]. This study involves human participants and was approved by the Ethics Committee of Renji Hospital (KY2020-055). To assess the effectiveness of specified inhibitors on HCC organoids, logarithmically growing pellets, containing 50,000 each, were resuspended in organoid media and subsequently plated onto a 24-well plate. DAPT (10 μM), ruxolitinib (10 μM) or their combination was added to the medium and their inhibitory effects on the formation of HCC organoids were observed under the microscope.

### Transmission electron microscope

The morphology of cells on carriers was visualized using a transmission electron microscope (TEM) (Hitachi S3400N, Hitachi). Briefly, the carriers were fixed with 2.5% glutaraldehyde at 4 °C overnight, and then dehydrated in a series of ethanol solutions (50, 75, 90, 95, and 100%). The resulting samples were dried, sputter-coated with gold, and observed using a TEM at a working voltage of 15 kV.

### Cells immunofluorescence (IF)

After washing with PBS, cell samples were fixed with 4% paraformaldehyde (PFA), and then blocked and permeabilized in PBS containing 5% goat serum and 0.1% Triton-X-100. For IF, cells were incubated with primary antibodies as follows: anti-E-cadherin (Cell Signaling Technology, USA), anti-Ki67, anti-TOM20 (Beyotime Biotechnology, China), anti-AFP (Proteintech, USA), anti-DARS2 (Proteintech, USA), anti-COX IV (Beyotime Biotechnology, China), anti-COX I (Beyotime Biotechnology, China), anti-P-STAT3 (Beyotime Biotechnology, China), anti-P-JAK1 (Beyotime Biotechnology, China), anti-Notch1 (Abcam, England) and anti-Jagged1 (Proteintech, USA). After 24 h, the cells were incubated in appropriate secondary antibodies for 1 h at room temperature in the dark. The secondary antibodies used for IF were as follows: Alexa fluor 488 donkey anti-mouse IgG, Alexa fluor 488 donkey anti-rabbit IgG, Alexa fluor 594 donkey anti-rabbit IgG, and Alexa fluor 594 donkey anti-mouse IgG (Yeasen Biotechnology, China). The nuclei were stained with DAPI (Beyotime Biotechnology, China). We used an Olympus FV3000 confocal microscope to obtain the images.

### Immunohistochemical (IHC) staining

Formalin-fixed paraffin-embedded samples were obtained from xenograft tumors. Paraffin-embedded tissues were cut into 4 μm sections on a microtome, mounted on glass microscope slides, and stored at room temperature. Then, samples were probed with antibodies anti-COX IV (Beyotime Biotechnology, China), anti-COX I (Beyotime Biotechnology, China). Following incubation with primary and secondary antibodies, positive cells were visualized using DAB^+^ as a chromogen.

### Mitochondrial transmembrane potential experiment (JC-1 staining)

Membrane permeability JC-1 staining (Yeasen Biotechnology, China) is widely used to monitor mitochondrial health. Cells were incubated with 1 × JC-1 at 37 °C for 30 min. Subsequently, cells were washed three times with PBS, and images were taken using a fluorescence microscope. Fluorescence intensity was detected using excitation and emission wavelengths of 550 nm and 600 nm, and excitation and emission wavelengths of 585 nm and 535 nm, respectively.

### Lentiviral vector transduction to generate stable NOTCH1 and STAT3 knockdown

Lentiviral plasmids containing a short hairpin RNA (shRNA) for genes encoding NOTCH1 and STAT3 and corresponding negative control were purchased from OBiO Technology Corp., Ltd (Shanghai, China). NM_017617.5 was used as the NOTCH1 transcript, and pCLenti-U6-shRNA-CMV-mCherry-F2A-BSR-WPRE was employed as the vector. For STAT3, NM_139276.3 was used as the transcript, and pSLenti-U6-shRNA-CMV-EGFP-F2A-Puro-WPRE was used as the vector. Before lentiviral vector transduction, 2 × 10^5^ HepG2 cells were seeded to 6-well plates, and transduction was conducted when cells reached 40–60% confluence. Briefly, vector control or shRNA (NOTCH1/STAT3) and 10 μg/mL polybrene (OBiO Technology) were added to HepG2 cells mixed with fresh 2 mL DMEM (10% FBS). After incubation for 48 h, infected cells were cultured in a selection medium containing 2 μg/mL blasticidin (NOTCH1 KD)/puromycin (STAT3 KD). Stably silenced HepG2 cells (NOTCH1 KD or STAT3 KD cell) were then maintained in DMEM with 2 μg/mL blasticidin/puromycin.

### Protein lysate preparation and western blotting

Cells were washed with PBS and lysed with RIPA buffer supplemented with complete protease inhibitor (Beyotime Biotechnology, China) and phosphatase inhibitor cocktails (Beyotime Biotechnology, China). All lysates were freshly prepared and processed with Super-PAGE™ Bis–Tris Gels Electrophoresis Systems (Epizyme Biomedical Technology, China). The source of antibodies is listed in Supplementary Information, Table 1.

### RNA sequencing and data processing

Total RNA was isolated from HepG2 and Hep3B cells in the presence and absence of HepLPCs for 48 h, 72 h, and from HepG2 and Hep3B cells treated with DAPT, Ruxo or both of them for 72 h. High throughput sequencing of mRNA was performed by Novogene (Beijing, China). Briefly, total RNA was extracted using Trizol Reagent (Vazyme). mRNA samples were purified using an NEB Next Poly(A) mRNA Magnetic Isolation Module Kit (NEB, E7490) according to the user’s manual. mRNA libraries were constructed using the Illumina TrueSeq mRNA sample preparation kit (Cat. No. RS-122–2101) following the manufacturer’s instructions. Library sequencing was performed on an Illumina NovaSeq 6000 instrument with 151 bp paired-end reads. Briefly, fastp software (v0.20.0) was used to trim adaptor, remove low-quality reads, and acquire high quality clean reads. STAR software (v2.7.9a) was used to align high-quality clean reads to the human reference genome (hg38). Feature Counts software (v2.0) was used to obtain the raw gene level mRNA read counts as the mRNA expression profile. DESeq2 software (v1.30.1) was used to normalize and calculate the fold change and *P* value between the two groups. Ensembl GTF gene annotation database (v104) was used to annotate the mRNA. Gene Ontology and KEGG pathway enrichment analyses were performed using cluster Profiler R package (v3.18.1) based on differentially expressed mRNAs. rMATS software (v4.1.1) was used to predict the alternative splicing events between the two groups. Bioinformatic data was analyzed by NewCore BioTech (Shanghai, China). The accession number for the gene arrays reported in this paper is GEO: GSE256376 and GSE256378.

### Xenografts

Animals were housed in micro-isolator cages measuring 30.5 cm × 19 cm × 14 cm. It included a wire rack for food and a water bottle. The animals were kept on a 12-h light/dark cycle. Mice were humanely killed using a CO_2_ chamber. All animals were manipulated and housed following the protocols approved by the Shanghai Model Organisms Center Inc., Institutional Animal Care and Use Committee (IACUC: 2020-0007-06). BALB/c nude mice (6–8 weeks, male) were randomly assigned to 2 groups. In the vehicle group, Huh7/Hep3B cells (5 × 10^6^ cells per mouse) were injected subcutaneously into the right armpit. In the mixed group, Huh7/Hep3B: HepLPCs with a 5:1 ratio (meaning 6 × 10^6^ cells per mouse) was injected subcutaneously into the right armpit, and the number of Huh7/Hep3B cells remained unchanged.

Liver orthotopic animal xenograft assays were conducted with 6-week-old male B-NDG. The mice were randomly divided randomly into two groups of six mice in each: Hep3B only, C-Hep3B (n = 6). Hep3B cells (5 × 10^5^ per mice) were mixed with HepLPC cells (1 × 10^5^ per mice) in a 5: 1 ratio, and indicated cells were injected under liver capsule to generate orthotopic implantation model. Mice in the control group (Hep3B only) were only implanted with Hep3B cells (5 × 10^5^ per mice). After 3 weeks, the mice were sacrificed, and liver samples were excised for further analysis.

For combined inhibition, Hep3B cells (5 × 10^6^ cells per mouse) were injected subcutaneously into the right front armpit of 6-week-old male BALB/c nude mice. Mice were randomly assigned to treatment (3 times/week) with vehicle, DAPT (20 mg/kg, oral gavage) [14], ruxolitinib (0.4 mg/mL, oral gavage) [15], or an inhibitor combination in which each compound was administered at the same dose and schedule as single agent.

For metabolite combination treatment, HepG2 cells (5 × 10^6^ cells per mouse) were injected subcutaneously into the right front armpit of 6-week-old male BALB/c nude mice. Mice were randomly assigned to treatment (3 times/week) with vehicle, SAM (100 mg/kg, intraperitoneal injection) [16], NA (50 mg/kg, intraperitoneal injection) [17], or a metabolite combination in which each compound was administered at the same dose and schedule as single agent.

### Untargeted metabolomics—materials and methods

The HepLPC-conditioned medium (FBS-free), MSC-conditioned medium (FBS-free), and primary hepatocyte-conditioned medium (FBS-free) were freeze-dried and resuspended with prechilled 80% methanol by well vortex. Then, the samples were incubated on ice for 5 min and centrifuged at 15,000*g* and 4 °C for 15 min. Part of the supernatant was diluted to the final concentration of 53% methanol by LC–MS grade water. The samples were subsequently transferred to a fresh Eppendorf tube and then centrifuged at 15,000*g* and 4 °C for 15 min. Finally, the supernatant was injected into the LC–MS/MS system analysis. UHPLC–MS/MS analyses were performed using a Vanquish UHPLC system (Thermo Fisher, Germany) coupled with an Orbitrap Q Exactive™ HF mass spectrometer (Thermo Fisher, Germany) in Novogene (Beijing, China). Raw data files generated by UHPLC–MS/MS were processed using Compound Discoverer 3.1 (CD3.1, Thermo Fisher) to perform peak alignment, peak picking, and quantitation of each metabolite. This section of the sequencing and analysis was accomplished by Novogene (Beijing, China).

### Statistical analysis

All data are presented as mean ± SEM. Data were analyzed via two-sided *t* test for two groups, or via one-way or two-way ANOVA for multiple groups using Prism software (GraphPad 8, San Diego, CA, USA). Differences were considered statistically significant at *p* < 0.05 in all the experiments.

## Results

### HepLPCs and their conditioned medium specifically suppressed the proliferation of liver cancer cells

**Fig. 1:**
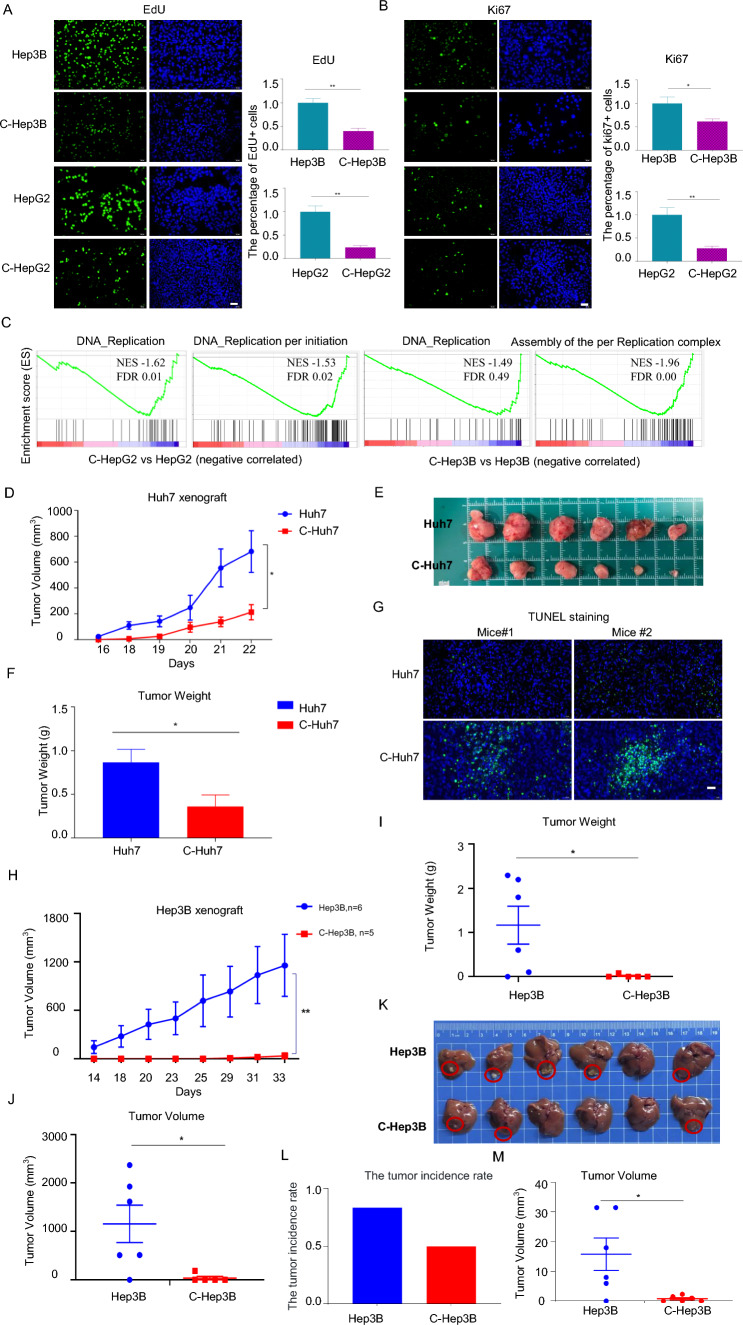
HepLPCs inhibited HCC proliferation in vitro and in vivo. **A** EdU insertion experiment was used to detect the proliferation of HCC before and after co-culturing with HepLPCs, scale bars: 50 μm. The charts on the right side revealed that co-culturing with HepLPCs for 48 h significantly inhibited the proliferation of HepG2 and Hep3B cells. **B** Ki67 staining of HepG2 and Hep3B cells in the presence or absence of HepLPCs for 2 days, scale bars: 50 μm. The graphs on the right displayed the quantitative comparison of Ki67-positive cells between the two groups. **C** GSEA indicating that DNA replication pathway and replication-per-initiation gene sets were downregulated in HepG2 (left) and Hep3B (right) cells co-cultured with HepLPCs compared with HepG2 and Hep3B only, *n* = 2. **D** Tumor volumes of Huh7 tumor xenografts in BALB/c nude mice 22 days after injecting the vehicle or HepLPCs, respectively, *n* = 6. **E** The bright field diagram shows the tumor volumes of the two groups, respectively. **F** Tumor weight of the two groups. **G** TUNEL assays were performed on formalin-fixed paraffin-embedded Huh7 xenografts after 22 days of treatment, scale bars: 20 μm. **H** Tumor volumes were measured after injecting Hep3B cells or a mixture with HepLPCs for 33 days in BALB/c nude mice. **I** The tumor weights of the two groups were measured. **J** Tumor volumes of the two groups were calculated. **K** Representative liver tissue images and tumor size of B-NDG mice orthotopically transplanted with Hep3B only or with a mixture of Hep3B and HepLPCs for 3 weeks (*n* = 6). The red circle marks the tumor formed by Hep3B cells. **L** Bar graph showing tumor formation efficiency of Hep3B cells and a mixture of Hep3B and HepLPCs in orthotopic models. **M** Tumor volumes of the two groups were calculated. Data are presented as mean ± SEM; Two-tailed Student's *t* test, *P < 0.05, **P < 0.01

To uncover HepLPCs–HCC cross-talk and the potential role of HepLPCs in the development of HCC, we optimized the co-culture condition of HepLPCs with HepG2 and Hep3B to sustain the viability for 4 days, adequate time to capture the long-term effects of HepLPCs on HCC cells. Unexpectedly, HepG2 or Hep3B cell count was evidently reduced after co-culturing with HepLPCs (S1A), and led to a significant reduction in the efficiency of EdU incorporation, as well as a decrease in the number of Ki67-positive cells (Fig. 1A, B). Gene set enrichment analyses (GSEA) of RNA-sequencing data from C-HepG2 and C-Hep3B cells showed the downregulation of a gene set related to DNA replication (Fig. 1C). Collectively, these data suggest that HepLPCs can suppress liver cancer cell growth in vitro.

To determine whether the inhibitory effects relied on direct cell–cell contact or was mediated by HepLPC-derived soluble factors, we cultured liver cancer cells in a conditional medium (CM) obtained from HepLPCs or a control medium (DMEM containing 10% FBS). After exposure to CM, the experimental group showed markedly decreased colony number compared with the control group (S1B). However, co-culturing with HepLPCs or exposure to the CM had little effect on the proliferation and morphology of normal cell lines 293T, L02, and bone marrow derived mesenchymal stem cells (BMSC) (S1C, D). We also conducted colony formation experiment to assess the effects of 293T, L02, and BMSC cells and their conditional medium on the proliferation of HepG2 and Hep3B cells. In contrast to HepLPC–CM, they had no significant inhibitory effect on cancer cell proliferation (S1E). Furthermore, we obtained a consistent result from liver cancer organoids treated with HepLPC–CM, showing that organoid formation was significantly inhibited (S1F). Notably, this specific inhibitory effect did not rely on cellular aging, autophagy, or apoptosis (S2). Marked changes in the malignant phenotype of HCC were observed following co-culture with HepLPCs, including significant alterations in cell viability, plate cloning efficiency, and invasion capacity (S3). These findings suggest that the interaction between HepLPCs and HCC cells initiated a complex series of molecular responses that culminate in the mitotic arrest.

### HepLPCs restricted the tumorigenicity of liver cancer cells in vivo

Next, we investigated whether our in vitro findings can be recapitulated in vivo. 5 × 10^6^ Huh7 cells alone or combined with 1 × 10^6^ HepLPCs were subcutaneously injected into mice, and tumorigenicity was determined. Consistent with the in vitro results, mice bearing Huh7 co-transplanted with HepLPCs showed a marked reduction in tumor volume (Fig. 1D), represented by the tumor bright field diagram (Fig. 1E). There was an obvious difference in tumor weight between the two groups, and the weight of tumors was reduced by approximately 51% in HepLPC-co-transplanted Huh7 (C-Huh7) relative to Huh7 only (Fig. 1F). Large necrotic areas were observed in TUNEL staining of residual tumors from mice receiving HepLPCs (Fig. 1G).

Similar results were also obtained from another in vivo experiment, in which we inoculated subcutaneously HepLPCs and Hep3B cells into nude mice. The tumor formation rate of Hep3B was 5/6, and Hep3B and HepLPCs was 1/5, respectively (Fig. 1H). Furthermore, the tumor weight and volume were much lower in experimental group compared with the control group (Fig. 1I, J). Moreover, HepLPCs effectively suppressed the proliferation of Hep3B cells within the orthotopic experimental model (Fig. 1K), resulting in a marked decrease in both tumor incidence rate (Fig. 1L) and tumor volume (Fig. 1M).

To investigate the persistence of HepLPCs during tumorigenesis, we co-implanted GFP labeled HepLPCs with an equal number of HepG2 cells into nude mice. Samples were collected after 2 days or 2 weeks to detect the remaining GFP-positive cells. The results showed that the number of GFP-positive HepLPCs in the experimental group is around 21 times higher on day 2 compared to that on day 14. Furthermore, after day 14, it was almost undetectable in experimental group (S4). These results suggest that HepLPCs may exert a therapeutic effect during the initial phases of tumor growth, possibly through paracrine signaling or direct cell–cell interactions that attenuate the malignant progression of HCC cells.

### Co-culturing with HepLPCs induced mitochondrial dysfunction in HCC

**Fig. 2:**
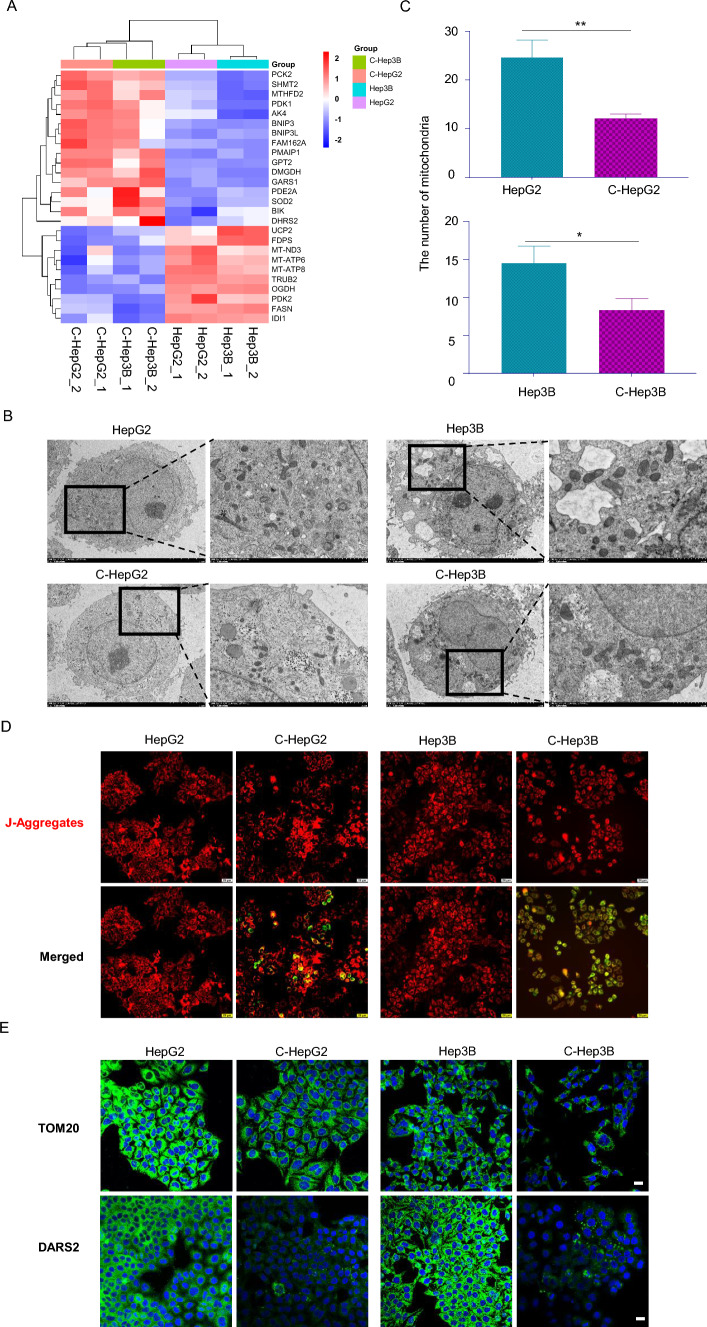

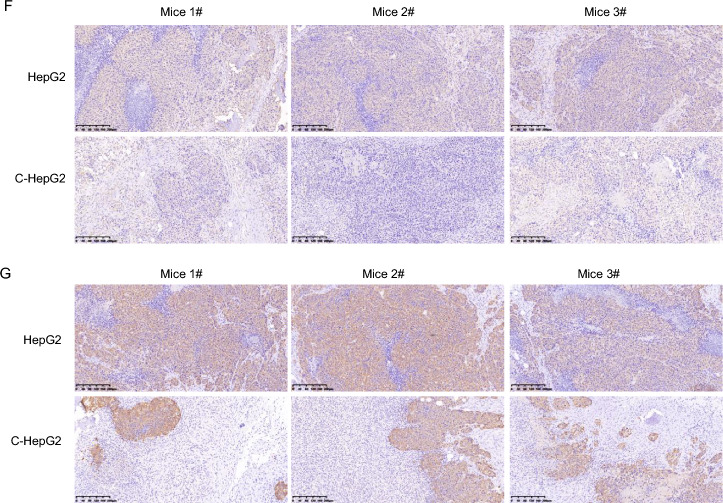
Co-culture with HepLPCs led to mitochondrial dysfunction in HCC cells. **A** A heatmap displayed that following co-cultivation, there were 26 differentially expressed genes associated with mitochondria in HCC after co-culturing with HepLPCs, of which 16 were upregulated and 10 were downregulated. **B** Electron microscopy was employed to investigate the number and integrity of mitochondria in HepG2 and Hep3B cells before and after co-culturing with HepLPCs. The scale bars for small images are 5 μm, and the scale bars for magnified images are 2 μm. **C** Quantify the number of mitochondria in the two groups. **D** JC-1 fluorescence assessment of mitochondrial function, scale bars: 50 μm. **E** Analysis of TOM20 and DARS2 expression before and after co-culturing with HepLPCs to assess mitochondrial function, scale bars: 20 μm. **F** Representative immunohistochemical images of COX I on formalin-fixed paraffin-embedded HepG2 xenografts after 14 days, scale bars, 200 μm. **G** Representative immunohistochemical images of COX IV on formalin-fixed paraffin-embedded HepG2 xenografts after 14 days, scale bars, 200 μm

Mitochondria are the powerhouses of the cell, providing the essential energy required for cell proliferation [18]. Our transcriptomic data revealed a downregulation of mitochondrial-related markers, including UCP2, MT-ND3, MT-ATP6, and MT-ATP8, in C-HepG2 and C-Hep3B cells (Fig. 2A). To further investigate this, we examined whether co-culturing impairs mitochondrial function. Using electron microscopy, we assessed the number and integrity of mitochondria in HepG2 and Hep3B cells in the presence and absence of HepLPCs (Fig. 2B). The results showed a decreased number of mitochondria (Fig. 2C) and abnormal mitochondrial morphology. In addition, mitochondrial membrane potential, assessed via JC-1 fluorescence, was reduced in HepG2 and Hep3B cells after co-culturing with HepLPCs, indicating mitochondrial dysfunction (Fig. 2D). Mitochondrial aspartyl-tRNA synthetase DARS2 deficiency leads to the suppression of the synthesis of respiratory chain subunits encoded by mitochondrial DNA (mtDNA), resulting in severe mitochondrial dysfunction [19]. Immunofluorescent staining for DARS2, alongside TOM20—a marker for the outer mitochondrial membrane (OMM)—demonstrated a significant impairment in mitochondrial function when HepLPCs were co-cultured with HepG2 or Hep3B cells (Fig. 2E). Immunohistochemical analysis revealed that HepLPCs suppressed the expression of COX I and COX IV (inner mitochondrial membrane proteins) in the HepG2 xenograft model (Fig. 2F, G). Collectively, these findings suggest that co-culturing with HepLPCs can lead to mitochondrial dysfunction in HCC cells.

### HepLPCs inhibited the Notch1 and JAK1-STAT3 pathway primarily due to mitochondrial dysfunction in HCC in vitro

**Fig. 3:**
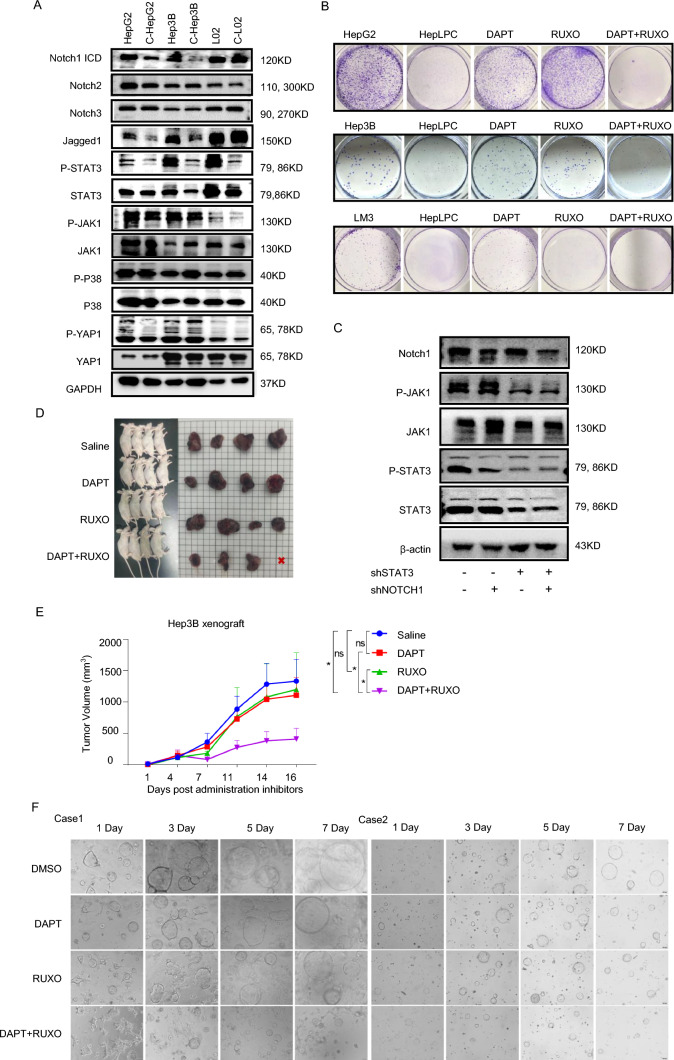
Combined Notch1 and JAK1/STAT3 inhibition constricted the proliferation of HCC cells. **A** Western blotting of Notch1 ICD, Notch2, Notch3, Jagged1, STAT3 (Tyr705) phosphorylation, JAK1 (Tyr1022/1023) phosphorylation, P38 (Thr180/Tyr182) phosphorylation, YAP1 (Ser127) phosphorylation, STAT3, JAK1, P38, and YAP1 in HepG2, Hep3B, and L02 cells in the presence or absence of HepLPCs for 72 h. GAPDH protein level was used as a loading control. **B** Short-term colony formation assays of a panel of liver cancer cell lines treated with DAPT (Notch inhibitor), ruxolitinib (STAT3 inhibitor), or their combination for 7 days. **C** Western blotting of designated proteins after shRNA-mediated knockdown of Notch1, STAT3, or both of them. **D** The bright field diagram shows the tumor volumes of the four groups, respectively. **E** The volumes of Hep3B tumor xenografts in BALB/c nude mice after treatment with vehicle, Notch inhibitor DAPT (20 mg/kg), STAT3 inhibitor ruxolitinib (0.4 mg/mL) or their combination for 16 days, *n* = 4. **F** Representative images of HCC organoids treated with DAPT (10 μM), ruxolitinib (10 μM), or the combination of both inhibitors for 7 days, scale bars: 100 μm. Data are presented as mean ± SEM; Two-tailed Student's *t* test, *P < 0.05, **P < 0.01

To unravel the molecular mechanisms underlying the inhibitory effects of HepLPCs on HCC cell proliferation, we analyzed the transcriptomic sequencing data and found a prevalence of abnormal expression of inflammation- and NOTCH-related genes among differential genes (S5A–C). Further screening on the related signaling pathways (S5D), the protein levels of Notch intracellular domain (NICD) and its ligand jagged1 were significantly reduced after co-culturing with HepLPCs. In addition, after co-culturing with HepLPCs, a marked decrease in the phosphorylation of STAT3 and JAK1 was observed, while the expression of Notch2, Notch3, P38 and YAP showed no significant changes (Fig. 3A). Representative immunofluorescent staining for P-STAT3 in HCC organoids exposed to HepLPC–CM confirmed the inhibition of P-STAT3 activity (S5E). Furthermore, we confirmed that HepLPCs inhibited the expression of both the Notch1 and JAK1–STAT3 signaling pathways in HepG2 xenografts (S6). These findings indicate that HepLPCs can inhibit the Notch1 and JAK1-STAT3 signaling in HCC cells. To determine whether combined inhibition of the Notch and JAK/STAT pathways could mimic the effects of HepLPCs, HCC cells were treated with 10 μM DAPT and 10 μM ruxolitinib (Ruxo) to inhibit the Notch1 and STAT3 signaling pathways, respectively. Consistent with the inhibitory effects observed with HepLPCs on HCC cells, the combination treatment significantly impaired the colony formation of various liver cancer cell lines compared to single treatments (Fig. 3B). Knockdown of Notch1 and STAT3 in HepG2 cells using shRNA further confirmed these effects (Fig. 3C).

Based on these in vitro findings, we proceeded to evaluate the synergistic effects of DAPT and ruxolitinib in vivo. Human Hep3B cells were subcutaneously injected into immunocompromised mice, and the xenografts were treated with either vehicle or a combination of DAPT (20 mg/kg) and ruxolitinib (0.4 mg/mL) for 14 days. Consistent with the in vitro results, the combined inhibition of Notch1 and STAT3 induced a more potent delay in tumor growth and reduced tumor volume (Fig. 3D, E). Two HCC-derived organoid models also demonstrated that the co-inhibition of Notch1 and STAT3 synergistically and significantly inhibited the formation of organoids (Fig. 3F). Gene ontology (GO) analysis revealed a noticeable downregulation of proliferation-related pathways (S7). Collectively, these data highlight the potent anti-tumor effects of the simultaneous inhibition of the Notch1 and JAK1/STAT3 pathways.

**Fig. 4:**
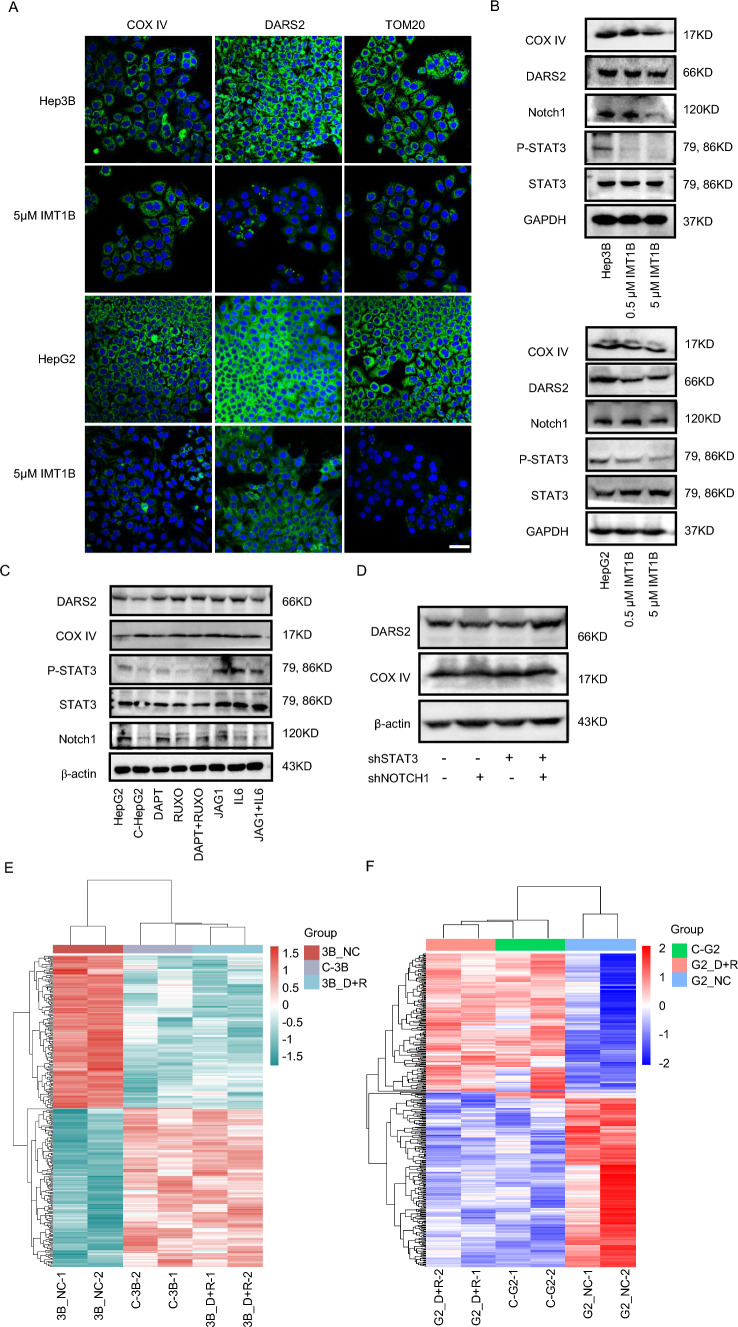
Mitochondrial dysfunction leads to the combined inhibition of Notch1 and STAT3 signaling in HCC cells. **A** Immunofluorescence staining for COX IV, DARS2, and TOM20 in HepG2 and Hep3B after treated with 5 μM IMT1B for 72 h, scale bars: 50 μm. **B** Western blotting of Notch1 ICD, STAT3 (Tyr705) phosphorylation, STAT3, DARS2, and COX IV in HepG2 and Hep3B cells treated with 0.5 μM, 5 μM IMT1B for 72 h. GAPDH protein level was used as a loading control. **C** Western blotting for DARS2, COX IV, Notch1 ICD, STAT3 (Tyr705) phosphorylation, and STAT3 in HepG2 after simultaneous inhibition or activation of Notch1 and JAK1/STAT3 signaling pathways. **D** Western blotting for DARS2, and COX IV in HepG2 after shRNA-mediated knockdown of Notch1, STAT3, or both of them. **E**, **F** Transcriptome analysis of HepG2 and Hep3B cells treated with HepLPC-CM, 10 μM DAPT, and 10 μM ruxolitinib, and their combination. Heatmap revealing a similar gene expression signature in HCC co-culturing with HepLPCs and HCC exposed to the combination of Notch inhibitor DAPT and STAT3 inhibitor ruxolitinib, compared with negative control, *n* = 2

To explore whether mitochondrial dysfunction could lead to the concurrent inhibition of the Notch1 and STAT3 signaling pathways, we first confirmed that the treatment of HCC cells with 5 μM ITM1B induced mitochondrial functional damage (Fig. 4A). The IMTs efficiently disrupt mtDNA transcription in a reconstituted recombinant system, causing a dose-dependent inhibition of mtDNA expression and oxidative phosphorylation (OXPHOS) in cell lines [20]. Subsequently, we assessed the changes in Notch1 and STAT3 levels following the induction of mitochondrial damage using ITM1B. The expression levels of DARS2 and COX IV indicated significant mitochondrial dysfunction, which was accompanied by a notable decrease in Notch1 expression and STAT3 phosphorylation levels in HepG2 and Hep3B cells (Fig. 4B). However, the concurrent inhibition of the Notch1 and STAT3 signaling pathways did not result in significant mitochondrial dysfunction, suggesting that the changes observed in the Notch1 and STAT3 signaling pathways are likely to be downstream effects of mitochondrial dysfunction (Fig. 4C, D). Interestingly, HCC co-cultured with HepLPCs and HCC exposed to the combination of Notch inhibitor DAPT and STAT3 inhibitor ruxolitinib exhibited a similar gene expression signature (Fig. 4E, F). These findings collectively suggest that HepLPCs primarily suppress HCC proliferation by inducing mitochondrial dysfunction, which in turn leads to the combined inhibition of the Notch1 and STAT3 signaling pathways.

### HepLPC-derived metabolites, SAM and NA, disrupted mitochondrial function and inhibited Notch1/STAT3-dependent liver cancer growth

Our subsequent investigation aimed to identify the principal factors derived from HepLPCs that regulate the proliferation of HCC. Given that co-cultivation with HepLPCs resulted in mitochondrial dysfunction in HCC cells and considering the intimate link between mitochondria and cellular metabolism, we concentrated on metabolically related candidate molecules.

**Fig. 5:**
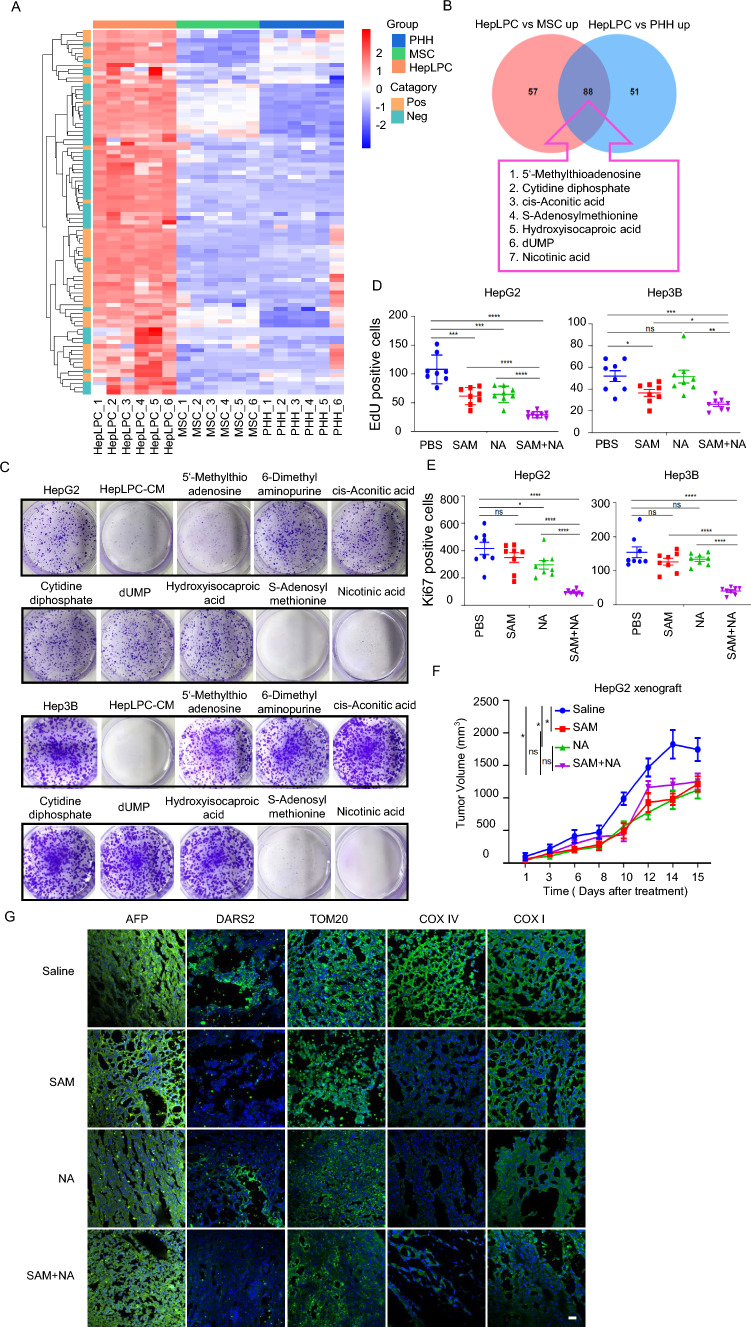
HepLPC-derived SAM and NA impaired mitochondrial function, inhibiting the progression of HCC. **A** Heatmap visualizing 88 dominantly differential metabolites in HepLPCs compared with MSC and PHH, *n* = 6. **B** Venn diagram showing the overlap of HepLPCs with MSCs and HepLPCs with PHH. Among the 88 overlapping upregulated metabolites, seven targetable metabolites were identified. **C** Short-term colony formation assays of HepG2 and Hep3B cells treated with indicated concentrations of candidate metabolites. **D** EdU insertion experiment was conducted to assess the proliferation of HepG2 and Hep3B cells following treatment with SAM, NA and their combination for 2 days. The results showed that treatment with SAM, NA alone, or in combination significantly inhibited the proliferation of liver cancer cells and exhibited synergistic effects. **E** Ki67 staining of HepG2 and Hep3B cells in the presence or absence of SAM, NA and their combination for 2 days. The results demonstrated that the combination of SAM and NA markedly suppressed the proliferation of liver cancer cells and exhibited synergistic effects. **F** The volumes of HepG2 xenografts in BALB/c nude mice after treatment with vehicle, SAM (100 mg/kg), NA (50 mg/kg) or their combination for 15 days, *n* = 6. **G** Representative microscopic images of AFP, DARS2, TOM20, COX IV and COX I showing the synergistic effect of the combination of SAM and NA on formalin-fixed paraffin-embedded HepG2 xenografts after 15 days of treatment, scale bars, 20 μm

An unbiased liquid chromatograph–mass spectrometry-based (LC–MS-based) metabolomic analysis revealed that HepLPCs contained 88 metabolites with higher specific content compared with MSC and primary human hepatocytes (PHH) (Fig. 5A). Through a combination of literature review and analysis of the Human Metabolome Database (HMDB), we ultimately identified 7 candidate metabolites (Fig. 5B). Notably, S-adenosylmethionine (SAM) and Nicotinic acid (NA) impaired colony formation in two HCC cell lines, while other metabolites did not exhibit such effects (Fig. 5C). The EdU insertion and Ki67 staining results indicated that 100 μM SAM, 100 μM NA, either alone or in combination, significantly suppressed the proliferation of liver cancer cells and exhibited synergistic effects (Fig. 5D, E).

To explore the in vivo tumorigenic effects of SAM and NA, HepG2 cells were transplanted into the nude mice to establish a subcutaneous tumor model. We demonstrated that SAM, NA, and their combination significantly reduced tumor burden in HepG2 cells (Fig. 5F), indicating that HepLPC-derived metabolites could effectively inhibit tumor growth in vivo. To assess whether SAM or NA induced mitochondrial dysfunction, we measured the expression of AFP, DARS2, COX IV, TOM20 and COX I using immunofluorescence staining on HepG2 xenografts (Fig. 5G). The results showed a synergistic response to the combination of SAM (100 mg/kg) and NA (50 mg/kg). These findings suggest that HepLPC-derived metabolites, specifically SAM or NA, alone or in combination, play a significant role in inducing mitochondrial dysfunction and inhibiting cell proliferation.

**Fig. 6:**
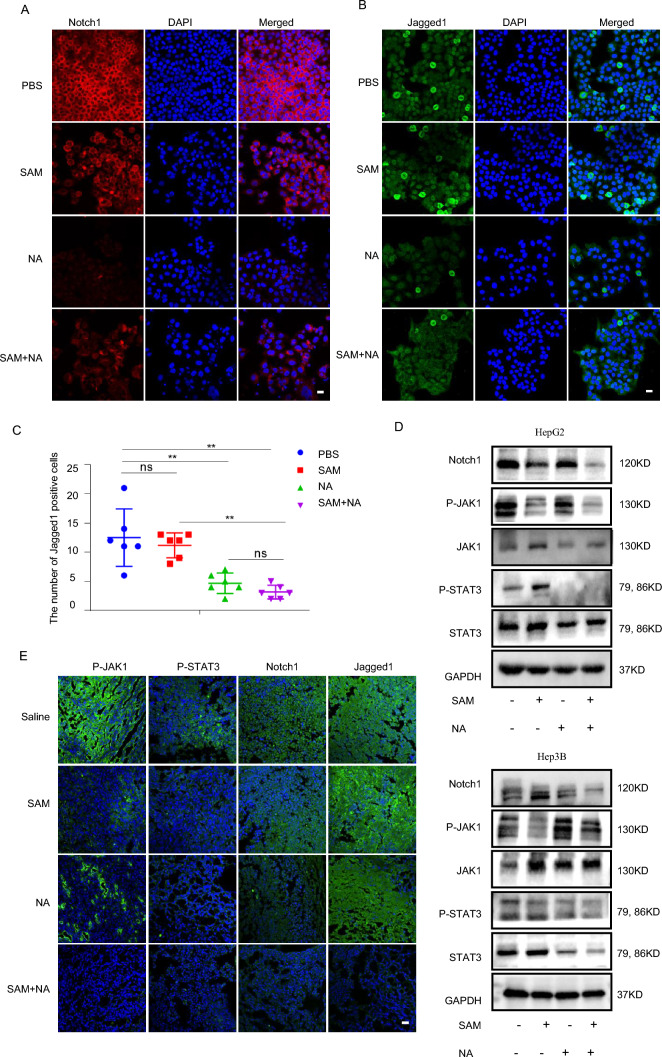
HepLPCs paracrine metabolites (NA and SAM) suppressed Notch1 and JAK1/STAT3 pathways to attenuate liver cancer progression. **A** Representative images for Notch1 expression were captured from HepG2 cells treated with SAM (100 μM), NA (100 μM), or their combination for 72 h scale bars: 20 μm. **B** Immunofluorescence staining for Jagged1 (green) was performed on HepG2 cells treated with SAM (100 μM), NA (100 μM) or their combination for 72 h, scale bar: 20 μm. **C** Quantification of Jagged1-positive cells (B). **D** HepG2 and Hep3B cells were treated with SAM (100 μM), NA (100 μM), or their combination for 72 h before western blotting. **E** Representative microscopic images of P-JAK1, P-STAT3, Notch1 and Jagged1 showing the synergistic effect of the combination of SAM and NA on formalin-fixed paraffin-embedded HepG2 xenografts after 15 days of treatment, scale bars, 20 μm. Data are presented as mean ± SEM; Two-tailed Student's *t* test, *P < 0.05, **P < 0.01

We also found that SAM and NA, alone or together, dramatically reduced the expression of Notch1 and its ligand Jagged1 in HepG2 cells (Fig. 6A–C). The phosphorylation levels of STAT3 and JAK1 were also reduced in the presence of SAM and NA (Fig. 6D). The immunofluorescence staining results from the HepG2 xenograft provide further evidence that the combined treatment of SAM and NA results in the suppression of both the Notch1 and JAK1–STAT3 signaling pathways (Fig. 6E). Taken together, our findings show that SAM and NA together induced sustained mitochondrial dysfunction and proliferation arrest through the co-inhibiting Notch1 and JAK1/STAT3 signaling pathways.

## Discussion

Our previous studies have identified the distinct therapeutic role of liver progenitor cells in liver injury, attributed to their robust paracrine function. In light of this, we explored the impact of HepLPCs on the growth of liver cancer cells. Here we found that HepLPCs significantly curtailed the proliferation and malignant behavior of liver cancer cells in both in vivo and in vitro settings through inducing mitochondrial dysfunction in these cancer cells. The underlying mechanism appeared to involve the suppression of HCC proliferation through the dual inhibition of the Notch1 and JAK1/STAT3 signaling pathways. This study revealed that HepLPCs might initiate this process by producing the metabolites SAM and NA.

There is an existing body of literature on the study of SAM and NA in the field of cancer research. Earlier studies have shown that SAM exert a potent anti-tumor effect in mouse models of HCC by acting as a methyl donor to modify H3K9me3 [21] and inhibiting the oncogene S100A11 [16]. SAM was also reported to prevent CCl4-induced cirrhosis [22, 23], inhibit tumor growth, and induce apoptosis in HCC [24, 25]. On the other hand, NA had been shown to improve the survival of rats with thioacetamide-induced liver fibrosis by attenuating the pro-oxidant process [26]. When combined with NAMPT inhibitor, NA maintains its strong therapeutic efficacy against tumors while significantly reducing toxicity in normal tissues[17]. It significantly inhibited the development of pre-neoplastic lesions in the early stages of HCC and induced mitochondria-mediated apoptosis in HCC cell lines [27]. Thus, it can be concluded that both SAM and NA supplementation could decelerate the progression of HCC, which align with the conclusion of this study that HepLPC-conditioned medium (HepLPC–CM), rich in SAM and NA, inhibits the proliferation of liver cancer cells.

SAM and NA are influential metabolites known to regulate the Notch1 and STAT3 signaling pathways, which play crucial roles in cellular development and homeostasis. The process of methylation, modulated by SAM, is particularly pertinent to the certain genomic regions has been found to correlate with the expression of the NOTCH1 gene [28]. Moreover, SAM's influence on cancer-related pathways can have downstream effects on the cell cycle and apoptosis, potentially leading to a reduction in the expression of the STAT3 protein [29]. This implies that SAM may impact the balance of cell growth and death mechanisms within the cellular microenvironment. On the other hand, Sirtuin3 (Sirt3), a nicotinamide adenine dinucleotide-dependent enzyme, regulates astrocyte activation by attenuating Notch1 signaling, thereby contributing to the inflammatory response following status epilepticus [30]. This suggests that NA, through its involvement in NAD^+^ metabolism and histone acetylation, can also modulate the activity of the Notch1 pathway. Collectively, SAM and NA, through their interactions with the epigenome and cellular metabolism, can exert regulatory effects on the Notch1 and STAT3 signaling pathways.

The JAK1/STAT3 signaling pathway plays a multifaceted role in cellular processes that are fundamental to the initiation and progression of HCC [31, 32]. Preclinical studies have demonstrated that the simultaneous targeting of these pathways can trigger autophagy and apoptosis in human malignant glioma cells [33, 34] and exert a synergistic suppression on the growth of lung cancer cells [35]. The co-inhibition of Notch1 and JAK1/STAT3 has also been shown to disrupt the proliferation, migration, and invasion of glioblastoma cells [34] and to inhibit the epithelial–mesenchymal transition [36]. Although the therapeutic benefits of co-inhibiting Notch1 and JAK1/STAT3 pathways have been noted in other diseases, their role in HCC has not been well-defined. Our findings provide evidence that this dual approach to pathway inhibition could be a novel and effective strategy in the treatment of liver cancer.

## Supplementary Information

Below is the link to the electronic supplementary material.

Supplementary file1 (PDF 2446 KB)
