# Supplementary material for: Inhibition of liver cancer cell growth by metabolites S-adenosylmethionine and nicotinic acid originating from liver progenitor cells
Source: J Gastroenterol. 2025 Feb 28;60(6):754–69. doi: 10.1007/s00535-025-02226-y (PMC12095412; doi:10.1007/s00535-025-02226-y)
Supplement: Supplementary file 1 — Supplementary file1 (PDF 2446 KB) [file 535_2025_2226_MOESM1_ESM.pdf]

Supplementary material

Inhibition of Liver Cancer Cell Growth by Metabolites S-adenosylmethionine and Nicotinic acid Originating from Liver Progenitor Cells

Wen-Ming Liu[1,2,3,4#], Cai-Yang Chen[1#], Hong-Qian Ma[1,3,4#], Qiu-Qiu Zhang[1,3,4], Xu Zhou[1], Yu-Ling Wu[1], Wei-Jian Huang[1], Xiao-Shu Qi[1], Yu-Xin Zhang[1,3,4], Dan Tang[1,3,4], Han-Yong Sun[5], Hong-Ping Wu[6], Ying-Fu Jiao[1,3,4], Zhi-Ying He[2]*, Wei-Feng Yu[1,3,4]* and He-Xin Yan[1,3,4]*

[1] *Department of Anesthesiology, Renji Hospital, Shanghai Jiaotong University School of Medicine, Shanghai, China.*

[2] *Institute for Regenerative Medicine, Shanghai East Hospital, School of Life Sciences and Technology, Tongji University, Shanghai, China.*

[3] *Key Laboratory of Anesthesiology (Shanghai Jiao Tong University), Ministry of Education, Shanghai, China.*

[4] *Shanghai Engineering Research Center of Peri-operative Organ Support and Function Preservation (20DZ2254200), Shanghai, China.*

[5] *Department of Liver Surgery, Renji Hospital, Shanghai Jiaotong University School of Medicine, Shanghai, China.*

[6] *Department of Laboratory Medicine, Eastern Hepatobiliary Surgery Hospital, Shanghai, China.*

[#]These authors contributed equally to this work.

*Corresponding author. Zhiying He, Weifeng Yu and Hexin Yan.

E-mail addresses:

zhiyinghe2002@163.com,

ywf808@yeah.net,

hexinyw@163.com.

Supplementary methods

HepLPC-conditioned medium collection

After 48 h of plating, conditional medium (CM) was collected from 1.5×10^5-2×10^5 HepLPC cells plated in a 6-cm dish with 2 ml of DMEM with penicillin/streptomycin. CM was centrifuged at 5,000 r.c.f. at 4 °C for 20 min, aliquoted, and stored at -80 °C. Then, 10% FBS was added to CM for treating cells. For all of these conditions, cell numbers were counted using a hemocytometer for normalization. In addition, a cell-free medium was used to assess background absorbance.

Cellular aging β-galactosidase staining

Cellular aging β-galactosidase staining kit was purchased from Beyotime Biotechnology. Staining was performed in accordance with the User's manual.

Live/dead viability assay

Cell viability was measured using the LIVE/DEAD® Viability/Cytotoxicity Kit for mammalian cells (Invitrogen).

Cell counting kit-8 (CCK-8) assay

To assess cell proliferation, 5×10^3 cells per well were seeded in 96-well plates containing conditioned medium for 24 h, 48 h, and 72 h. Then, the medium was replaced with a medium containing 10% (v/v) CCK-8 (Beyotime Biotechnology) and incubated for 1 h. The absorbance was read at 450 nm using a multimode reader Synergy 2 (BioTek).

Xenografts

Animals were housed in micro-isolator cages measuring 30.5 cm × 19 cm × 14 cm. It included a wire rack for food and a water bottle. The animals were kept on a 12-hour light/dark cycle. Mice were humanely killed using a CO_2 chamber. All animals were manipulated and housed following the protocols approved by the Shanghai Model

Organisms Center Inc., Institutional Animal Care and Use Committee (IACUC: 2020-0007-06). 6-week-old male BALB/c nude mice were randomly assigned to 2 groups. In the vehicle group, HepG2 cells (5×10^6 cells per mouse) were injected subcutaneously into the right front armpit. In the mixed group (HepG2: fluorescently labeled HepLPCs) with a 1:1 ratio (1×10^7 cells per mouse) was injected subcutaneously into the right front armpit. After 2 days and 2 weeks, samples were collected for GFP histological staining to assess the residual status of HepLPCs.

Immunohistochemical (IHC) staining

Formalin-fixed paraffin-embedded samples were obtained from xenograft tumors. Paraffin-embedded tissues were cut into 4 μm sections on a microtome, mounted on glass microscope slides, and stored at room temperature. Then, samples were probed with antibodies against GFP (Abcam, England), anti-AFP (Proteintech, USA), anti-P-STAT3 (Beyotime Biotechnology, China), anti-Notch1 (Abcam, England) and anti-Jagged1 (Proteintech, America). Following incubation with primary and secondary antibodies, positive cells were visualized using DAB^+ as a chromogen. In addition, H&E staining and TUNEL staining were done according to the manufacturer's protocol.

Statistical analysis

All data are presented as mean ± SEM. Data were analyzed via two-sided t-test for two groups, or via one-way or two-way ANOVA for multiple groups using Prism software (GraphPad 8, San Diego, CA, USA). Differences were considered statistically significant at $p < 0.05$ in all the experiments.

Antibody	Company	Cat.No	Source	Dilution
Notch1	Abcam	ab27526	Rabbit	1-1000
Phospho-STAT3 (Tyr705) Rabbit Monoclonal Antibody	Beyotime	AF1276	Rabbit	1-1000
Stat3 Rabbit Monoclonal Antibody	Beyotime	AF1492	Rabbit	1-1000
Jagged1 Monoclonal antibody	Proteintech	66890-1-Ig	Mouse	1-1000
E-Cadherin (4A2) Mouse mAb	CST	14472	Mouse	1-1000
p38 MAPK (D13E1) XP® Rabbit mAb	CST	8690	Rabbit	1-1000
Phospho-p38 MAPK (Thr180/Tyr182) (D3F9) XP® Rabbit mAb	CST	4511	Rabbit	1-1000
Phospho-Jak1 (Tyr1022/1023) Rabbit Polyclonal Antibody	Beyotime	AF5857	Rabbit	1-1000
JAK1 Monoclonal antibody	Proteintech	66466-1-Ig	Mouse	1-1000
YAP (D8H1X) XP® Rabbit mAb	CST	14074	Rabbit	1-1000
YAP1 (Phospho-Ser127) Rabbit mAb	SAB signalway	14199	Rabbit	1-1000
Phospho-ULK1 (Ser555) (D1H4) Rabbit mAb 5869	CST	5869	Rabbit	1-1000
ULK1 (D8H5) Rabbit mAb 8054	CST	8054	Rabbit	1-1000
Beclin-1 (D40C5) Rabbit mAb 3495	CST	3495	Rabbit	1-1000
Phospho-Beclin-1 (Ser93) (D9A5G) Rabbit mAb 14717	CST	14717	Rabbit	1-1000
p53 Antibody	CST	9282	Rabbit	1-1000
p16 INK4A (E6N8P) Rabbit mAb	CST	18769	Rabbit	1-1000
p21 Waf1/Cip1 (12D1) Rabbit mAb	CST	2947	Rabbit	1-1000
PARP	CST	9542	Rabbit	1-1000
Cleaved PARP (Asp214) Antibody	CST	9541	Rabbit	1-1000
Caspase-3 Antibody	CST	9662	Rabbit	1-1000
Cleaved Caspase-3 (Asp175) Antibody	CST	9661	Rabbit	1-1000
Notch2 (D76A6) XP® Rabbit mAb	CST	5732S	Rabbit	1-1000
Notch3	Abcam	ab23426	Rabbit	1-1000
Notch4	Abcam	ab33163	Rabbit	1-1000
STAT1 Rabbit Monoclonal Antibody	Beyotime	AG3318	Rabbit	1-1000
Phospho-Stat1 (Tyr701) (D4A7) Rabbit mAb	CST	7649S	Rabbit	1-1000
DARS2 Polyclonal antibody	Proteintech	13807-1-AP	Rabbit	1-1000
AFP Polyclonal antibody	Proteintech	14550-1-AP	Rabbit	1-500
COX IV Rabbit Monoclonal Antibody	Beyotime	AG8011	Rabbit	1-1000
COX I Rabbit Polyclonal Antibody	Beyotime	AF6546	Rabbit	1-1000
TOM20 Polyclonal antibody	Proteintech	11802-1-AP	Rabbit	1-1000
GAPDH Mouse Monoclonal Antibody	Beyotime	AF0006	Mouse	1-5000
β-Actin Mouse Monoclonal Antibody	Beyotime	AF0003	Mouse	1-5000

Anti-mouse IgG, HRP-linked Antibody	CST	7076	Mouse	1-5000
Anti-rabbit IgG, HRP-linked Antibody	CST	7074	Rabbit	1-5000

Supplementary table 1. Antibody list.

Abbreviation	Full name
HepLPCs	Human hepatocyte-derived liver progenitor-like cells
HCC	hepatocellular carcinoma
SAM	S-adenosylmethionine
NA	Nicotinic acid
MSCs	mesenchymal stem cells
Sirt3	Sirtuin3
LPC	liver progenitor cell
JAK1	Janus tyrosine kinase 1
STAT3	Signal transducer and activator of transcription 3
STAT1	Signal transducer and activator of transcription 1
DMEM	Dulbecco's Modified Eagle Medium
CO_2	carbon dioxide
STR	short tandem repeat
CM	Conditional medium
CCK8	Cell counting kit-8
DAPI	2-(4-amidinophenyl)-6-indolecarbamidine dihydrochloride
PBS	phosphate-buffered saline
DAPT	N-[N-(3, 5-difluorophenacetyl)-l-alanyl]-s-phenylglycinet-butyl ester
RUXO	Ruxolitinib
TEM	Transmission Electron Microscope
OXPHOS	oxidative phosphorylation
TOM20	translocase of the outer membrane 20
IgG	Immunoglobulin G
JC-1	5,5',6,6'-tetrachloro1,1',3,3'-tetramethylbenzimidazolylcarbocyanine iodide
DARS2	Mitochondrial aspartyl-tRNA synthetase
COX IV	cytochrome c oxidase IV
COX I	cytochrome c oxidase I
AFP	Alpha-fetoprotein
RIPA	Radioimmunoprecipitation Assay
P38	p38 mitogen-activated protein kinases
YAP1	Yes-associated protein 1
PARP	Poly(ADP-ribose) polymerase
ULK1	UNC-52-like kinase 1

GAPDH	Glyceraldehyde-3-phosphate dehydrogenase
HRP	Horseradish peroxidase
hg38	human reference genome
IHC	Immunohistochemical
IF	Immunofluorescence
EdU	5-ethynyl-2'-deoxyuridine
GSEA	Gene set enrichment analyses
DNA	deoxyribonucleic acid
RNA	Ribonucleic acid
TUNEL	Terminal deoxynucleotidyl transferase dUTP nick-end labeling
GFP	Green fluorescent protein
BMSC	bone marrow mesenchymal stem cell
OMM	outer mitochondrial membrane
NICD	Notch intracellular domain
PHH	primary human hepatocyte
HMDB	Human Metabolome Database
CCl4	Carbon tetrachloride

Supplementary table 2. List of abbreviations.

S1. HepLPCs-derived conditional medium (CM) specifically inhibited the proliferation of HCC.

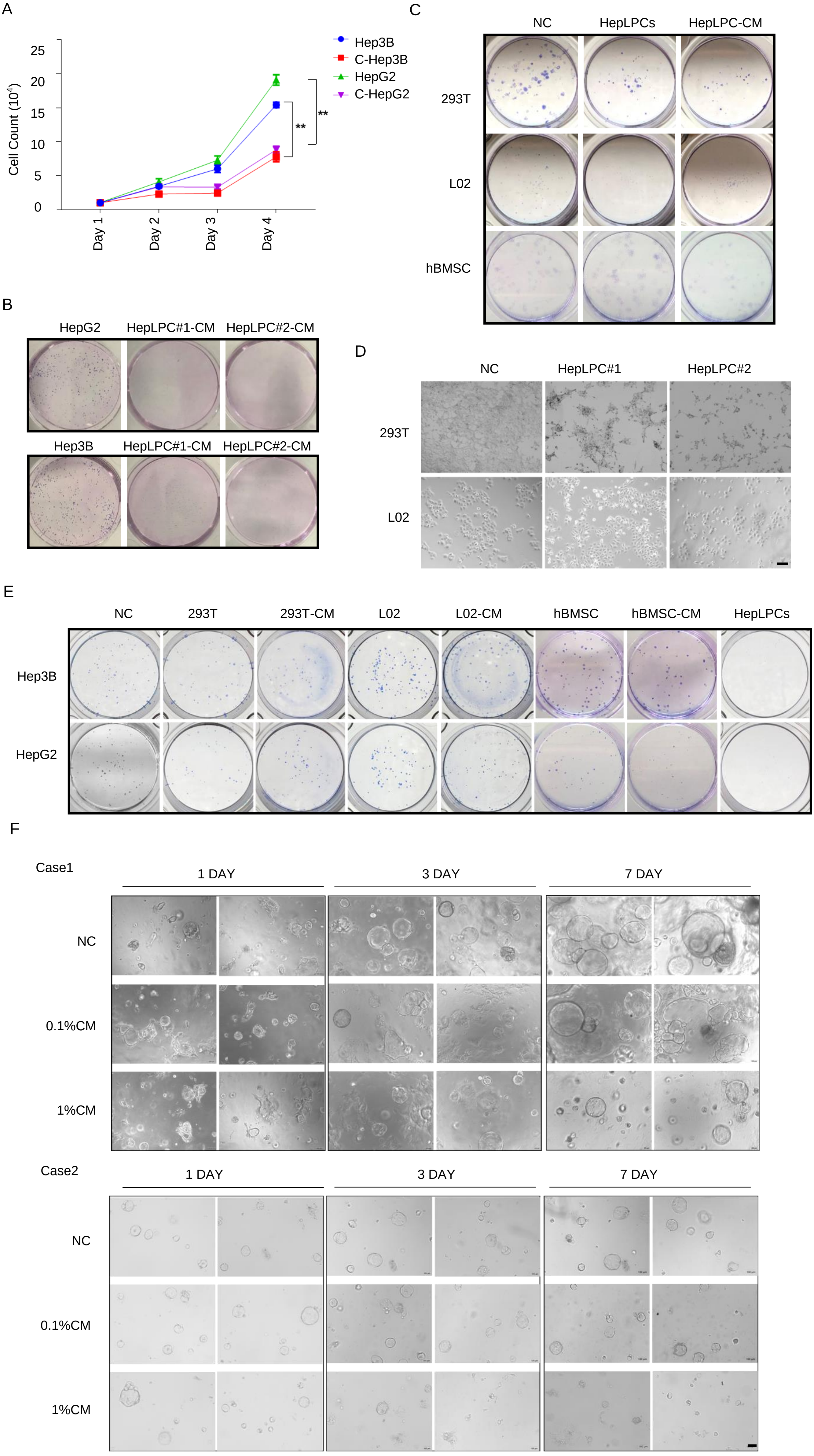

S1. HepLPCs-derived conditional medium (CM) specifically inhibited the proliferation of HCC. A. HepG2 and Hep3B were cultured in the presence or absence of HepLPCs, and cell numbers were counted. B. Short-term colony formation assays of HepG2 and Hep3B cells co-cultured with HepLPCs originated from different clones. C. Crystal violet staining of 293T, L02, and BMSC cells treated with blank control, HepLPCs, and HepLPC-CM for 7 days. D. Representative images of 293T and L02 after co-culturing with HepLPCs derived from 2 clones. Scale bars, 100 μm. E. Crystal violet staining of HepG2 and Hep3B cell colonies treated with 293T, L02, BMSC, and their conditioned medium for 7 days. HepLPC-CM treatment was a positive control. F. Representative images of HCC organoids treated with HepLPC-CM for 7 days. Scale bars, 100 μm.

S2. Co-culturing with HepLPCs did not affect the aging, autophagy, or apoptosis of HCC cells.

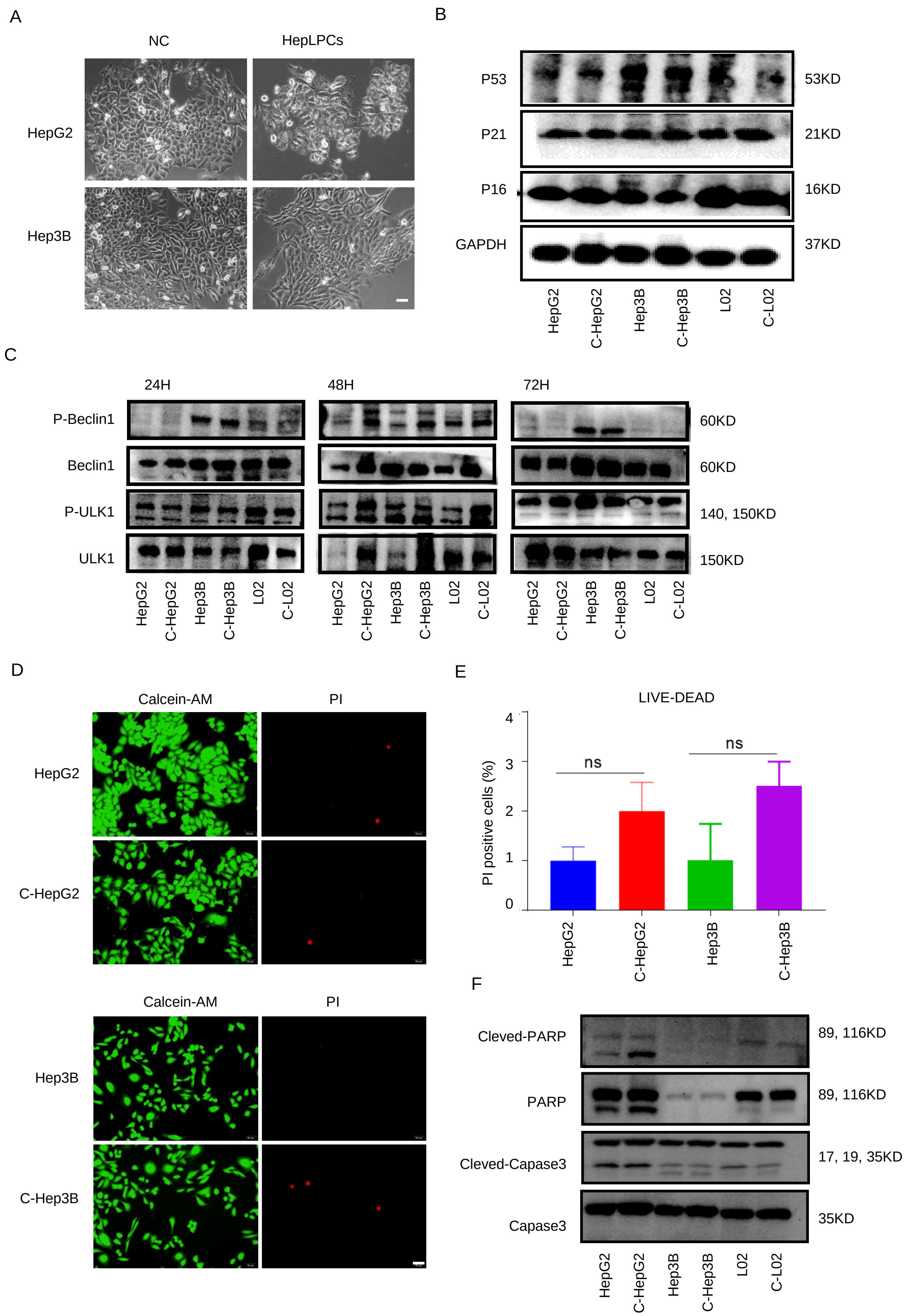

S2. Co-culturing with HepLPCs did not affect the aging, autophagy, or apoptosis of HCC cells. A. Representative microscopic images of β-Gal staining of HepG2 and Hep3B cells before and after co-culturing with HepLPCs. Scale bars, 50 μm. B. Western blotting of senescence-related markers in HepG2, Hep3B, and L02 cells in the absence or presence of HepLPCs. C. HepG2, Hep3B, and L02 cells were co-cultured with HepLPCs before western blotting with autophagy-related antibodies. D. HepG2 and Hep3B cells were subjected to Calcein-AM/PI assay to detect apoptotic phenotype. Scale bars, 50 μm. E. PI-positive cells (D) were quantified. F. Treated HepG2, Hep3B and L02 cells were subjected to western blotting to measure the expression of apoptosis-associated proteins.

S3. Alteration in the malignant behavior of HCC after co-culturing with HepLPCs.

A

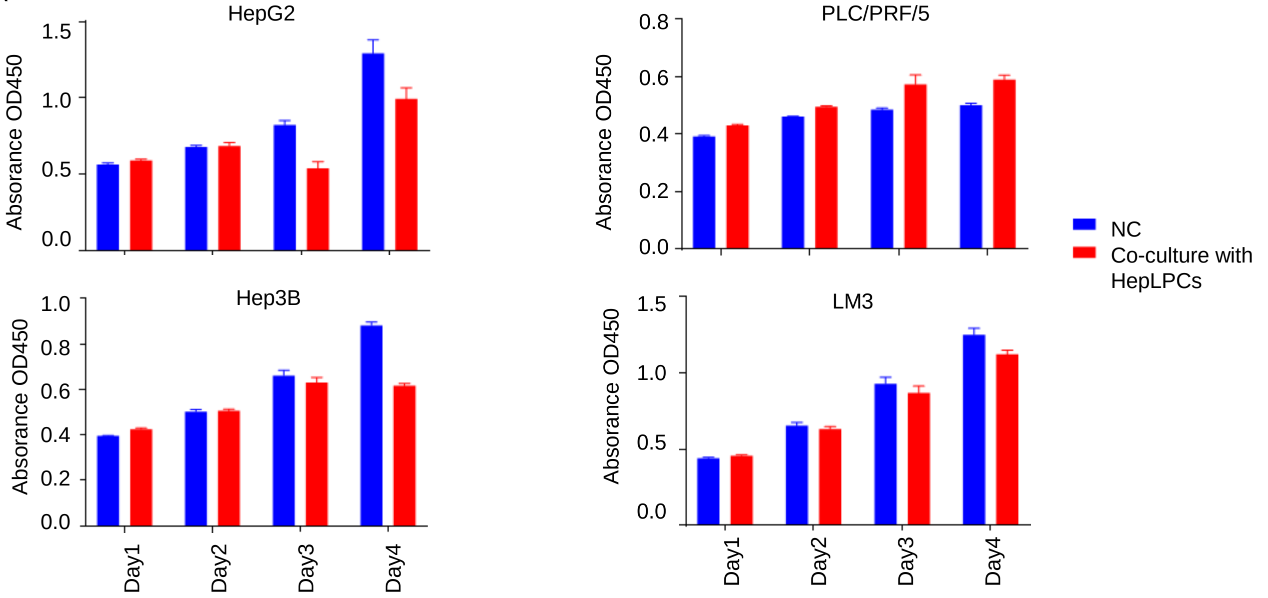

B

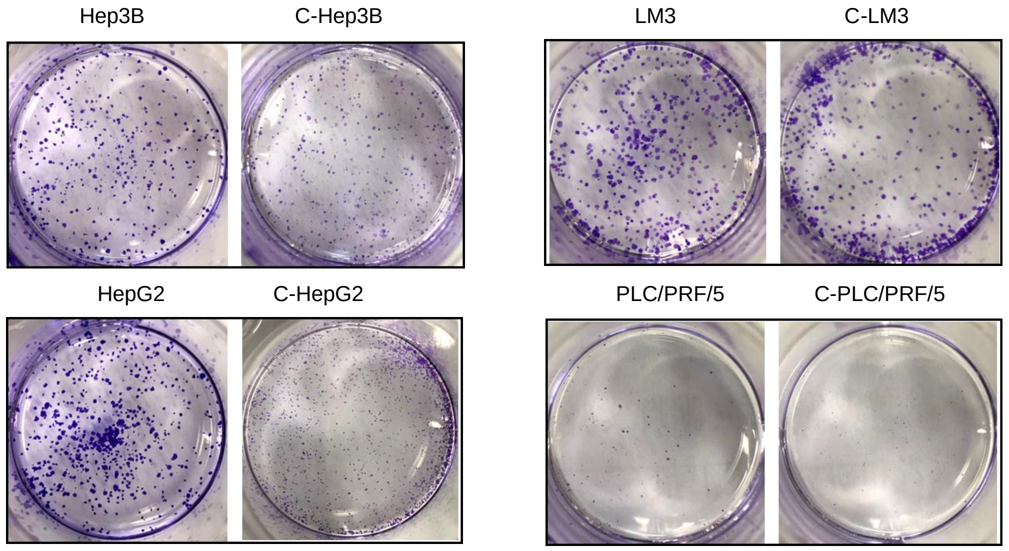

C

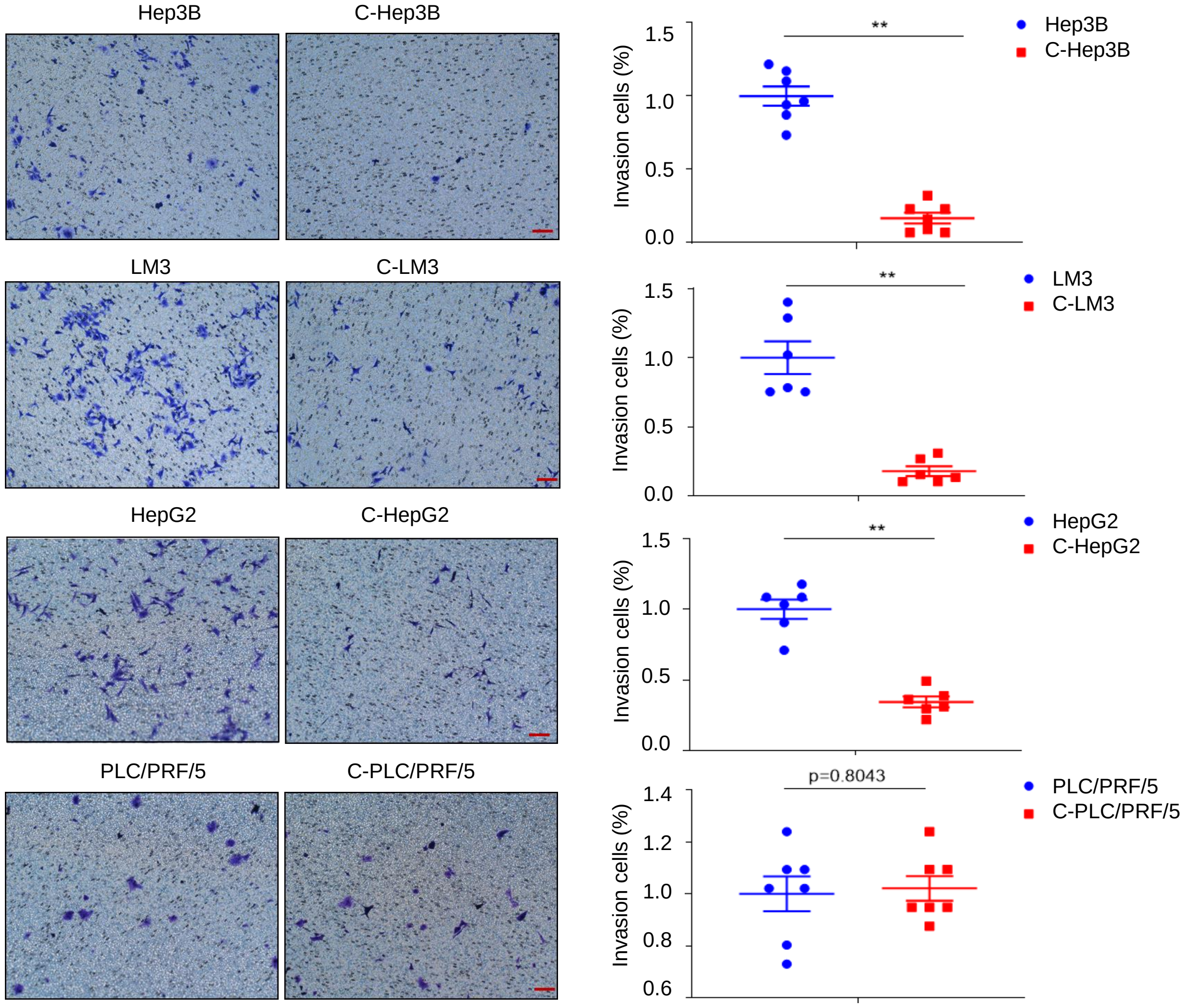

S3. Alteration in the malignant behavior of HCC after co-culturing with HepLPCs. A. Cell viability was measured using the CCK-8 assay. B. Crystal violet staining after replanting pretreated cells. C. Cell invasion assay was used to evaluate the invasive ability of cells.

S4. Role of HepLPCs in liver cancer progression.

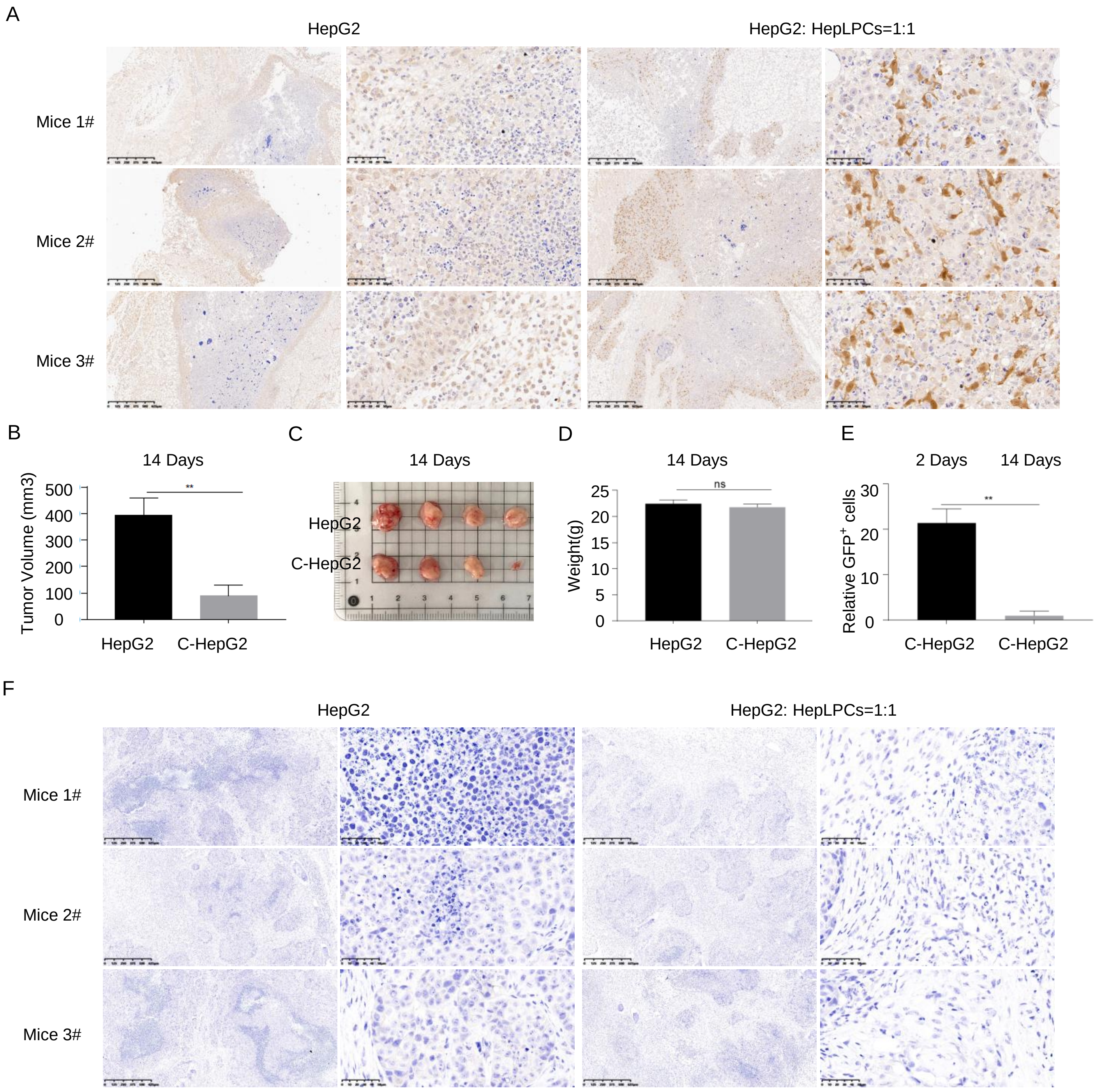

S4. Role of HepLPCs in liver cancer progression. A. Representative images of GFP on formalin-fixed, paraffin-embedded HepG2 xenografts from mice sacrificed after 2 days. The scale bars for small images are 625 μm, and the scale bars for magnified images are 50 μm. B. The volumes of HepG2 xenografts in BALB/c nude mice following treatment with vehicle, mixed with green fluorescent protein labeled HepLPCs for 14 days. C. Bright field diagram of *ex vivo* tumors from mice sacrificed after 14 days. D. Body weight measurements of mice bearing HepG2 xenograft tumors after 14 days. E. Quantification of GFP-positive cells. F. Representative images of GFP on formalin-fixed, paraffin-embedded HepG2 xenografts of mice sacrificed after 14 days. The scale bars for small images are 625 μm, and the scale bars for magnified images are 50 μm.

S5. Screening for JAK/STAT and Notch family members was conducted in a panel of liver cancer cell lines.

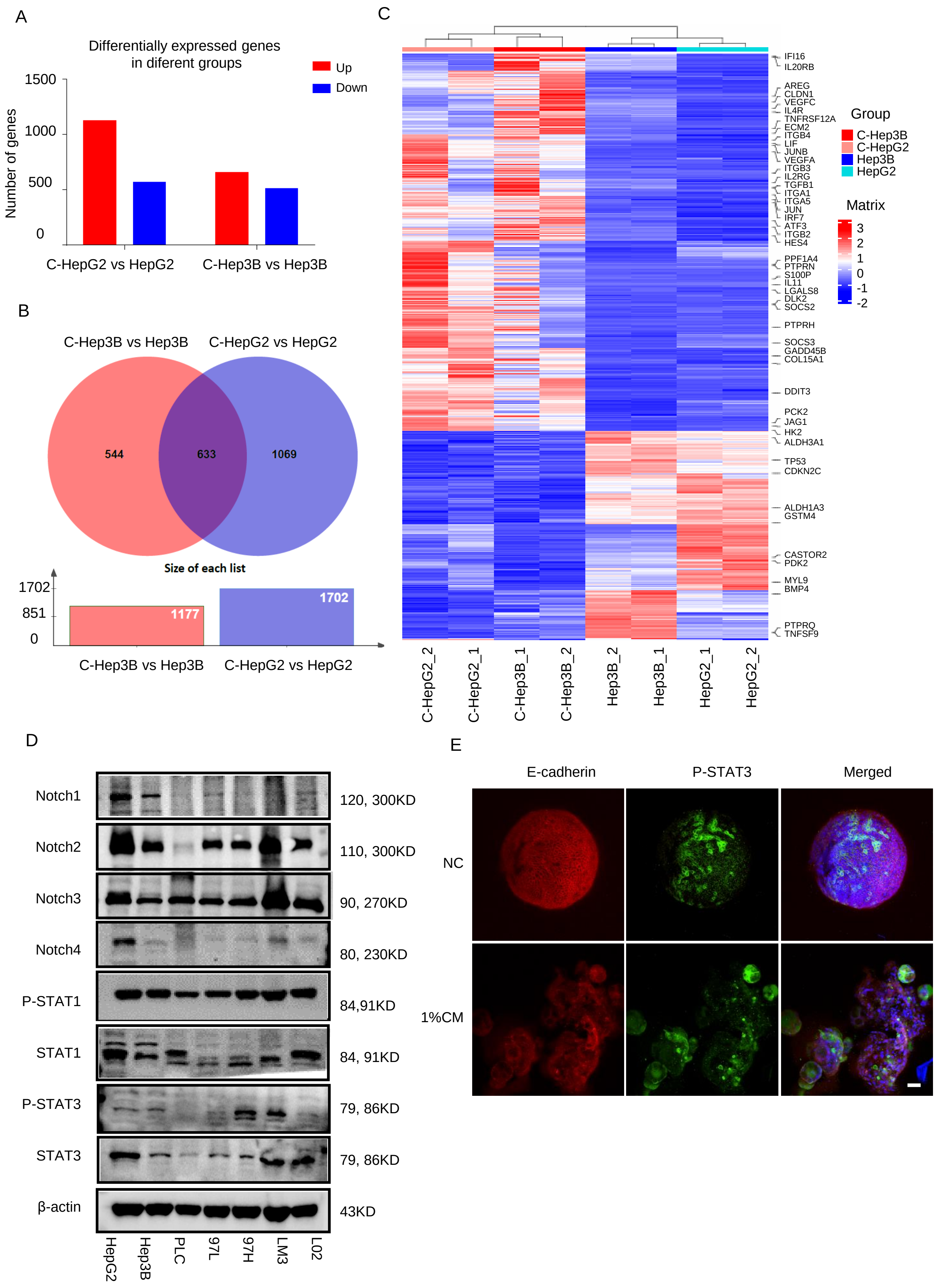

S5. Screening for JAK/STAT and Notch family members was conducted in a panel of liver cancer cell lines. A. The number of differentially expressed genes in HepG2 and Hep3B cells after co-culturing with HepLPCs. C-HepG2 had 1130 genes with high expression and 572 genes with low expression compared with HepG2; Compared with Hep3B, C-Hep3B had 663 genes with high expression and 514 genes with low expression. B. The overlap of differentially expressed genes between C-HepG2 and HepG2 and between C-Hep3B and Hep3B. C. Cluster analysis of 663 differentially expressed genes. D. Western blotting showing the expression of the Notch family and JAK/STAT members in a panel of liver cancer cell lines. E. Representative immunofluorescent staining for P-STAT3 in HCC organoids treated with HepLPC-CM. Scale bars, 50 μm.

S6. HepLPCs inhibit the expression of the Notch1 and JAK1-STAT3 signaling pathways *in vivo*.

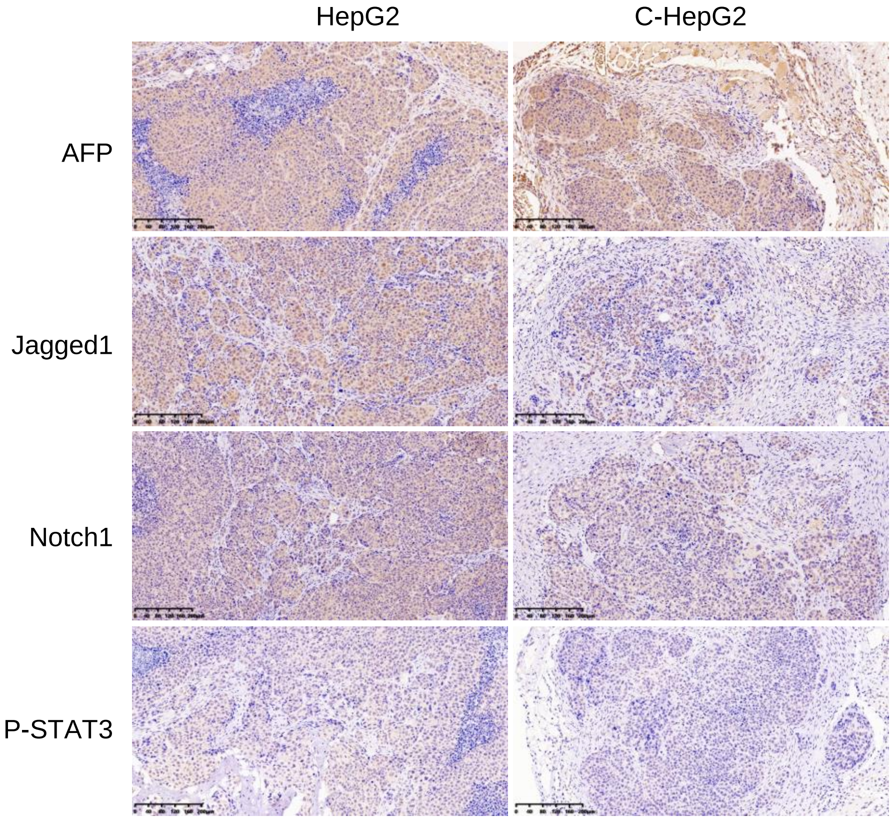

S6. HepLPCs inhibit the expression of the Notch1 and JAK1-STAT3 signaling pathways in HepG2 xenograft. Representative images of AFP, Notch1, Jagged1 and P-STAT3 expression in formalin-fixed, paraffin-embedded HepG2 xenografts from mice sacrificed after 14 days. The scale bars in the images are 200 μm.

S7. Combined inhibition of Notch1 and JAK1/STAT3 pathways blocked the proliferation of HCC cells.

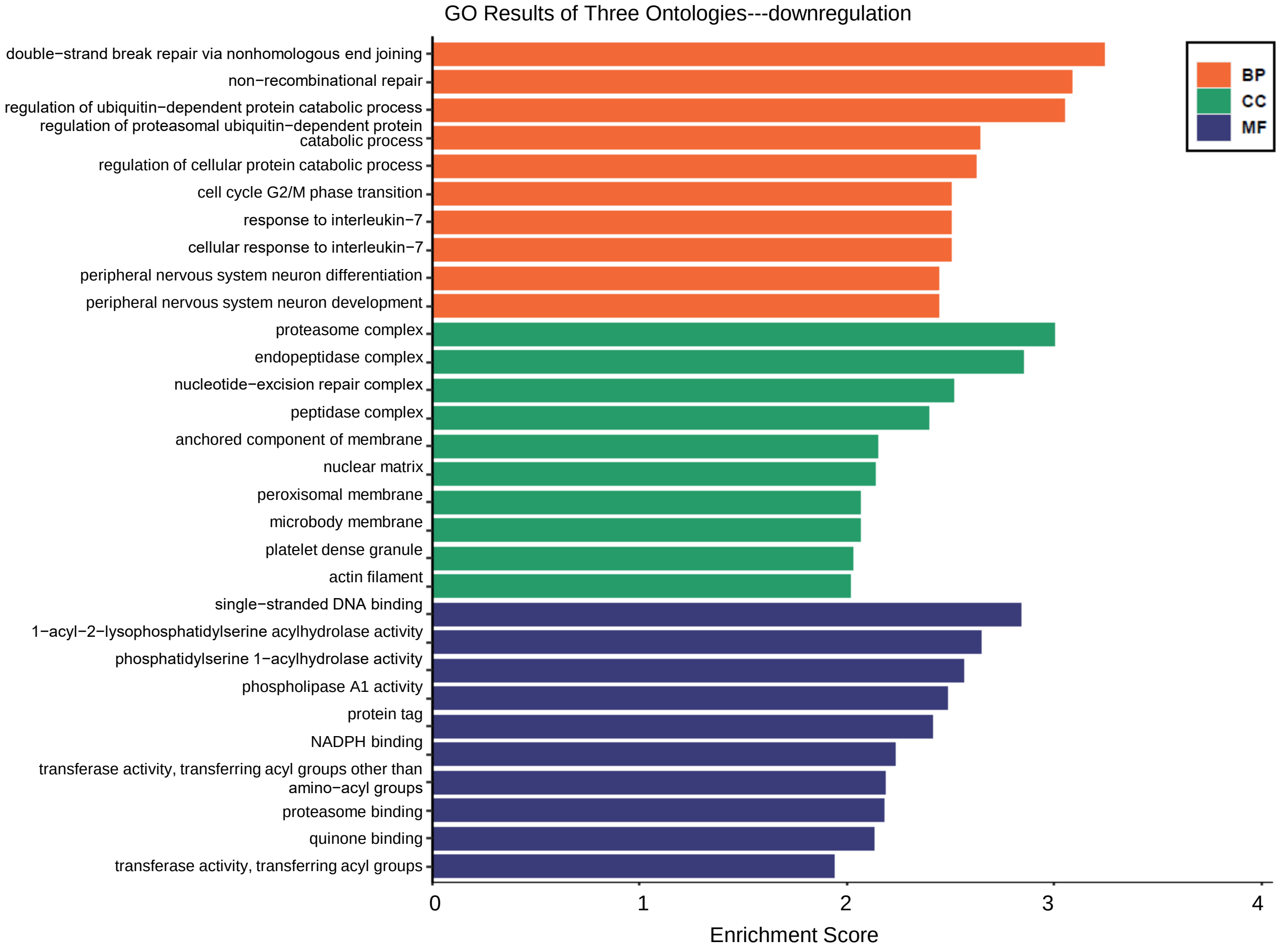

S7. Combined inhibition of Notch1 and JAK1/STAT3 pathways blocked the proliferation of HCC cells. Gene Ontology analysis of genes downregulated in Hep3B cells subjected to treatment with the combination of 10 μM DAPT and 10 μM ruxolitinib for 72 hours.
